# Disruption of *Mycobacterium avium* subsp. *paratuberculosis*-specific genes impairs *in vivo* fitness

**DOI:** 10.1186/1471-2164-15-415

**Published:** 2014-05-31

**Authors:** Joyce Wang, Justin R Pritchard, Louis Kreitmann, Alexandre Montpetit, Marcel A Behr

**Affiliations:** Department of Microbiology and Immunology, McGill University, 3775 University Street, Montreal, QC H3A 2B4 Canada; Department of Immunology and Infectious Diseases, Harvard School of Public Health, Boston, MA 02115 USA; Department of Medicine, McGill University, 1650 Cedar Avenue, Montreal, QC H3G 1A4 Canada; McGill University and Génome Québec Innovation Centre, 740 Dr. Penfield Avenue, Montreal, H3A 0G1 QC Canada; McGill International TB Centre, 1650 Cedar Avenue, Montreal, QC H3G 1A4 Canada

**Keywords:** *Mycobacterium avium*, *M. avium* subsp. *paratuberculosis*, Transposon insertion sequencing, Horizontal gene transfer, Mycobacterial pathogenesis

## Abstract

**Background:**

*Mycobacterium avium* subsp. *paratuberculosis* (MAP) is an obligate intracellular pathogen that infects many ruminant species. The acquisition of foreign genes via horizontal gene transfer has been postulated to contribute to its pathogenesis, as these genetic elements are absent from its putative ancestor, *M. avium* subsp. *hominissuis* (MAH), an environmental organism with lesser pathogenicity. In this study, high-throughput sequencing of MAP transposon libraries were analyzed to qualitatively and quantitatively determine the contribution of individual genes to bacterial survival during infection.

**Results:**

Out of 52384 TA dinucleotides present in the MAP K-10 genome, 12607 had a MycoMarT7 transposon in the input pool, interrupting 2443 of the 4350 genes in the MAP genome (56%). Of 96 genes situated in MAP-specific genomic islands, 82 were disrupted in the input pool, indicating that MAP-specific genomic regions are dispensable for *in vitro* growth (odds ratio = 0.21). Following 5 independent *in vivo* infections with this pool of mutants, the correlation between output pools was high for 4 of 5 (R = 0.49 to 0.61) enabling us to define genes whose disruption reproducibly reduced bacterial fitness *in vivo*. At three different thresholds for reduced fitness *in vivo*, MAP-specific genes were over-represented in the list of predicted essential genes. We also identified additional genes that were severely depleted after infection, and several of them have orthologues that are essential genes in *M. tuberculosis*.

**Conclusions:**

This work indicates that the genetic elements required for the *in vivo* survival of MAP represent a combination of conserved mycobacterial virulence genes and MAP-specific genes acquired via horizontal gene transfer. In addition, the *in vitro* and *in vivo* essential genes identified in this study may be further characterized to offer a better understanding of MAP pathogenesis, and potentially contribute to the discovery of novel therapeutic and vaccine targets.

**Electronic supplementary material:**

The online version of this article (doi:10.1186/1471-2164-15-415) contains supplementary material, which is available to authorized users.

## Background

*Mycobacterium avium* subspecies *paratuberculosis* (MAP) is an intracellular pathogen that causes Johne’s disease, a chronic (2 to 5 years) intestinal inflammation in cattle, sheep, goats and other ruminants [1]. When MAP is shed into the environment from an infected host, its survival is finite, with no evidence of bacterial replication [2], indicating that the definitive host of MAP is the ruminant species in which it has co-evolved. In contrast, the closely-related organism, *M. avium* subspecies *hominissuis* (MAH), is considered an environmental generalist, as it can be isolated and propagated in a variety of reservoirs, including water sources and biofilms [3–5]. How MAP has evolved into a professional pathogen remains largely unknown.

In other bacterial pathogens such as *Escherichia coli*, *Salmonella enterica*, *Shigella flexneri*, and *Yersinia enterocolitica*, the transfer of DNA from one organism to another member of a different species has been shown to contribute to the emergence of virulent strains. Interestingly, in many cases the transferred DNA contains clusters of genes known as pathogenicity islands that enable the recipient strain to adapt to the host environment [6–11]. In the case of MAP, the completion of the genome sequences of MAP K10 and MAH 104 has greatly enabled the derivation of an evolutionary model for the emergence of MAP [12] along with the identification of MAP-specific genomic islands that are absent in MAH [13] (MAP: [GenBank:AE016958] [14] and revised version [GenBank: SRR060191] [15]; MAH: [GenBank:CP000479] provided by the J. Craig Venter Institute). Although horizontal gene transfer (HGT) has been detected in mycobacteria [16–18], the functional consequence of acquiring these novel genetic elements is currently unknown in this genus. In this study, we wished to examine whether MAP-specific genomic fragments contribute to the survival of MAP within the host.

Mutagenesis-mediated approaches have been employed extensively with great success for the determination of conditionally essential genes in a number of bacterial pathogens including *Mycobacterium tuberculosis*, *Pseudomonas aeruginosa*, *Salmonella* species, *Vibrio cholerae*, and *Neisseria meningitidis*[19–27]. Previous studies employing the transposon (Tn) mutagenesis strategy have identified MAP genes involved in metabolism and host adaptation using selected, genetically-defined mutants [28–31]. These studies indicate the feasibility of mutagenizing this organism with the purpose of conducting unbiased, genome-wide scale screens of conditionally essential genes of MAP. In this study, we have used high-throughput Illumina sequencing to characterize transposon libraries and identify genes whose disruption is deleterious for *in vivo* survival. In particular, we were interested in whether MAP-specific genes (i.e. genes absent from MAH strains) were over- or under-represented in genes predicted to contribute to survival *in vitro* or *in vivo*. Our data suggest that MAP-specific genes are dispensable for *in vitro* survival yet over-represented in genes required for MAP persistence in the mouse model. These findings present a methodology that can be readily applied to selected experimental conditions, including infection of natural mammalian hosts of MAP.

## Results

### Generation of M. avium subsp. paratuberculosis K-10 transposon library

**Figure 1:**
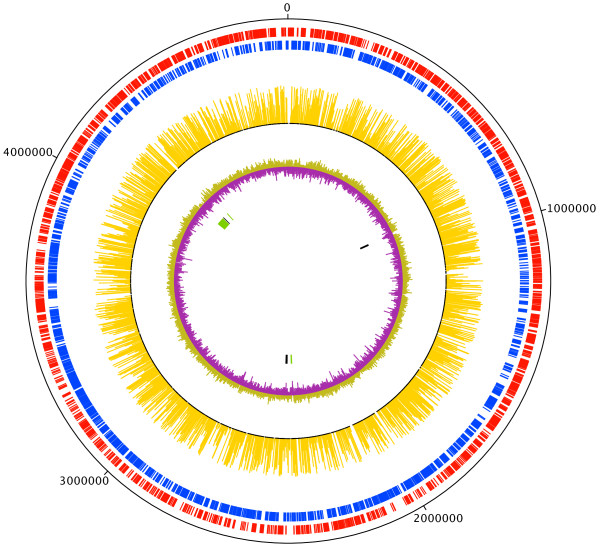
**Input reads mapped onto the** ***M. avium*** **subsp.** ***paratuberculosis*** **K-10 genome.** The amplitude of each peak corresponds to the number of sequence reads at a specified insertion site. The circles represent (from outer to inner): CDS on plus strand (red); CDS on minus strand (blue); transposon insertion reads (yellow);% GC plot (light brown = above average, purple = below average), and MAP-specific genomic islands in centre (LSP^P^4, 11, 12, 14, 15, 16 in alternating black and green blocks clockwise). CDS: coding DNA sequence.

Out of 52384 TA dinucleotides present in the MAP K-10 genome, 12607 were found to be targeted by the MycoMarT7 transposon in the input pool. We tallied TA positions that were aligned by ≥ 11 reads, resulting in 7784 unique disruptions. This corresponds to 2443 disrupted genes, or 56% of the 4350 genes in the MAP genome. The distribution of the mapped reads is shown in Figure 1.

### MAP-specific genes within Large Sequence Polymorphisms (LSP^P^s) are dispensable for in vitro survival

Of the 96 genes situated within 6 previously defined MAP-specific genomic islands (also known as large sequence polymorphisms, LSP^P^s ([13]), 82 were disrupted. Based on the proportion of genes disrupted in the entire genome (56%), the expected number of surviving LSP^P^ transposon mutants was 54. The difference between expected and observed surviving LSP^P^ mutants was significant by chi-squared testing as indicated in Table 1. The odds ratio of LSP^P^ disruption being associated with *in vitro* absence was calculated to be 0.21.

**Table 1:** **Summary of observed surviving LSP** ^**P**^ **mutants in the input pool**

Input			
Observed	Survived Tn mutants	Did not grow ***in vitro***	Total
LSP^P^	82	14	96
not LSP^P^	2361	1893	4254
Total	2443	1907	4350
Chi-square test	*p* = 5.1 × 10^-9^		

The chi-squared test was used to compare the difference between the observed and the expected distribution of LSP^P^ genes (expected values not shown). *p* value = 5.1 × 10^-9^.

### Mutants with disruption in MAP-specific genes were depleted after animal infection

As described in the Methods section, we generated 5 independent sets of output:input ratios for all sites. When these ratios were compared across experiments, a correlation coefficient of ~ 0.5 to 0.6 was seen when comparing the ratios from outputs 1-4; in contrast, output 5 data revealed a much lower correlation coefficient when compared to each of the other 4 experiments (Table 2). As a result, output 5 was removed from downstream analysis. Each gene was assigned an output to input ratio, and the median of these ratios was 1.44, 1.50, 1.42, and 1.30 for the 4 output pools. To tease out genes important for *in vivo* fitness, we examined genes, that when disrupted, resulted in mutants with an output to input ratios less than 0.1 × average median of that experiment, reasoning that these genes would represent priority candidates for future targeted investigation. We identified a total of 415 depleted mutants at this threshold; while the expected number of depleted LSP^P^ mutants was 14, we observed 26, demonstrating that the LSP^P^ genes were over-represented in genes important for *in vivo* fitness. We tested two additional thresholds, 0.2 × median and 0.05 × median, and the results are summarized in Table 3. The odds ratio of LSP^P^ gene disruption and *in vivo* depletion at 0.1 × median was 2.35. LSP^P^ genes that were depleted *in vivo* at 0.1 × median are listed in Table 4. Lists of depleted genes at various median thresholds are presented in Additional file 1.

**Table 2:** **Correlation coefficient between each set of output:input ratio**

	Ratio 1	Ratio 2	Ratio 3	Ratio 4	Ratio 5
Ratio 1	n.a.				
Ratio 2	0.61	n.a.			
Ratio 3	0.54	0.59	n.a.		
Ratio 4	0.50	0.56	0.49	n.a.	
Ratio 5	0.19	0.19	0.18	0.17	n.a.

n. a. = not applicable.

**Table 3:** **Summary of observed surviving LSP** ^**P**^ **mutants in the output pool**

Observed	Survived Tn	Depleted	Total
**Output - 0.2 × median**			
LSP^P^	49	33	82
not LSP^P^	1711	650	2361
Total	1760	683	2443
Chi-square test	*P* = 0.01		
**Output - 0.1 × median**			
LSP^P^	56	26	82
not LSP^P^	1972	389	2361
Total	2028	415	2443
Chi-square test	*P* = 0.0003		
**Output - 0.05 × median**			
LSP^P^	69	13	82
not LSP^P^	2137	224	2361
Total	2206	237	2443
Chi-square test	*P* = 0.06		

Summary of observed surviving LSP^P^ mutants in the output pool at different thresholds compared to expected values (values not shown), and the *p* value of corresponding chi-square test.

**Table 4:** **LSP** ^**P**^ **genes depleted** ***in vivo*** **(as defined in **[13]**)**

LSP^P^	Region description	Average ratio	Standard deviation	Predicted function
4	MAP0856c (MAPK_2912)	0.0843	0.0720	H.P.
4	MAP0862 (MAPK_2906)	0.0189	0.0378	H.P.
4	MAP0865 (MAPK_2903)	0.0000	0.0000	Cell division protein
11	MAP2148 (MAPK_1620)	0.0653	0.0640	Phage integrase
11	MAP2150 (MAPK_1618)	0.0827	0.0087	Transposase
11	MAP2154c (MAPK_1614)	0.1038	0.0153	H.P.
11	MAP2157 (MAPK_1611)	0.1216	0.0910	Transposase
11	MAP2158 (MAPK_1610)	0.1255	0.0333	H.P.
12	MAP2185c (MAPK_1583)	0.1410	0.1087	Amidohydrolase
12	MAP2194 (MAPK_1574)	0.0787	0.0703	Mce family protein
14	MAP3731c (MAPK_0037)	0.0302	0.0509	ABC transporter ATP-binding Protein
14	MAP3733c (MAPK_0035)	0.1041	0.0890	H.P.
14	MAP3735c (MAPK_0033)	0.0557	0.0533	ABC transporter ATP-binding Protein
14	MAP3741 (MAPK_0027)	0.0039	0.0005	H.P.
14	MAP3742 (MAPK_0026)	0.0265	0.0178	H.P.
14	MAP3745 (MAPK_0023)	0.0104	0.0154	H.P.
14	MAP3750 (MAPK_0018)	0.0111	0.0057	MmpS1 family protein
14	MAP3751 (MAPK_0017)	0.0746	0.0970	transmembrane transport protein, MmpL4_5
14	MAP3757c (MAPK_0011)	0.0923	0.0679	H.P.
14	MAP3759c (MAPK_3761)	0.0836	0.0106	Tranposase
14	MAP3760c (n. a.)	0.0400	0.0065	H.P.
14	MAP3763c (MAPK_3765)	0.0372	0.0267	PapA2_3
14	MAP3764c (MAPK_3766)	0.1181	0.1928	Pks2
15	MAP3776c (MAPK_3778)	0.1353	0.2561	ABC transporter periplasmic solute binding protein
16	MAP3816 (MAPK_3818)	0.0701	0.0757	Phage integrase
16	MAP3817c (MAPK_3819)	0.1251	0.0114	H.P.

LSP^P^ genes depleted *in vivo* with an average ratio of less than 0.1 × median (0.153). Gene annotation by Li *et al* shown in second column with Wynne *et al* annotation in brackets. H.P. = Hypothetical protein, Mce = Mammalian cell entry, Mmp = Membrane protein, Pap = Polyketide associated protein, Pks = Polyketide synthase, n. a. = not annotated.

### MAP “*in vivo*” essential genes and comparison with *M. tuberculosis* conditionally attenuated mutants

Among the 415 depleted mutants at 0.1 × median, 29 were undetectable in the output pool in all 4 mice analyzed. Compared to Tuberculist (http://tuberculist.epfl.ch/) [32] in which *in vitro* and *in vivo M. tuberculosis* essential genes are compiled [19–21], many orthologues of these MAP genes are also essential, either *in vitro* or *in vivo*, in *M. tuberculosis*. A list of these genes is presented in Table 5.

**Table 5:** **Genes absent from all 4 output pools**

Zero output	Mtb orthologue	Essentiality in H37Rv	Protein function in ***M. tuberculosis***
MAP0298 (MAPK_3470)	Rv1129c	Essential for growth of H37Rv on cholesterol *in vitro*	Probable transcriptional regulator protein
MAP0704 (MAPK_3064)	Rv3121	Essential for *in vitro* growth	Probable cytochrome P450 141 Cyp141
MAP0865 (MAPK_2903)	Rv0284	Essential for *in vitro* growth	ESX conserved component EccC3 ESX-3 type VII
MAP0908c (MAPK_2860)	Rv0966c	Non-essential for *in vitro* growth	Hypothetical protein
MAP0977 (MAPK_2791)	Rv1013	Required for growth in C57BL/6 J mouse spleen	Putative polyketide synthase Pks16
MAP1031c (MAPK_2737)	Rv2601	Non-essential for *in vitro* growth	Probable spermidine synthase SpeE
MAP1082c (MAPK_2686)	Rv1936	Non-essential for *in vitro* growth	Possible monooxygenase
MAP1195c (MAPK_2573)	Rv1467c	Non-essential for *in vitro* growth	Probable acyl-CoA dehydrogenase FadE15
MAP1236c (MAPK_2532)	Rv2938	Required for growth in C57BL/6 J mouse spleen	Probable daunorubicin-dim-transport integral membrane protein ABC transporter DrrC
MAP1576 (MAPK_2192)	Rv1866	Non-essential for *in vitro* growth	Hypothetical protein
MAP1584c (MAPK_2184)	Rv2897c	Essential for *in vitro* growth	Hypothetical protein
MAP1601 (MAPK_2167)	n.a.		
MAP1605c (MAPK_2163)	Rv1882c	Non-essential for *in vitro* growth	Probable short-chain type dehydrogenase/reductase
MAP1822c (MAPK_1946)	Rv3903c	Non-essential for *in vitro* growth	Hypothetical alanine and proline rich protein
MAP1835c (MAPK_1933)	Rv2110c	Essential for *in vitro* growth and encoded proteasome required for persistence in mice	Proteasome beta subunit PrcB
MAP1914 (MAPK_1854)	Rv2176	Non-essential for *in vitro* growth	Probable transmembrane serine/threonine-protein kinase L PknL (protein kinase L)
MAP2008 (MAPK_1760)	Rv2259	n.d.	S-nitrosomycothiol reductase MscR
MAP2385c (MAPK_1383)	Rv3543c	Essential for growth of H37Rv on cholesterol *in vitro*	Probable acyl-CoA dehydrogenase FadE29
MAP2439c (MAPK_1329)	Rv1321	Non-essential for *in vitro* growth	Hypothetical protein
MAP2582c (MAPK_1186)	Rv1866	Non-essential for *in vitro* growth	Hypothetical protein
MAP2964c (MAPK_0804)	Rv1701	Essential for *in vitro* growth	Probable integrase/recombinase
MAP3070 (MAPK_0698)	Rv3829c	Non-essential for *in vitro* growth	Probable dehydrogenase
MAP3131 (MAPK_0637)	Rv0450c	Essential for *in vitro* growth	Probable conserved transmembrane transport protein MmpL4
MAP3327c (MAPK_0441)	Rv3529c	Non-essential for *in vitro* growth	Hypothetical protein
MAP3352c (MAPK_0416)	Rv1358	n.d.	Probable transcriptional regulatory protein
MAP3420c (MAPK_0348)	Rv1705c	Non-essential for *in vitro* growth	PPE family protein PPE22
MAP3699c (MAPK_0069)	Rv0249c	Required for growth in C57BL/6 J mouse spleen	Probable succinate dehydrogenase [membrane anchor subunit]
MAP3951c (MAPK_3953)	Rv0457c	Non-essential for *in vitro* growth	Probable peptidase
MAP4117c (MAPK_4119)	Rv0645c	Non-essential for *in vitro* growth	Methoxy mycolic acid synthase 1

*M. tuberculosis* orthologues are assigned based on the KEGG database (Kyoto Encyclopedia Genes and Genomes; http://www.kegg.jp/[56]) and essentiality information is found on Tuberculist. Gene annotation by Li *et al* shown in first column with Wynne *et* al annotation in brackets. n.a. = not applicable, n.d. = no data, PPE = Pro-Pro-Glu.

## Discussion

To unambiguously investigate the essentiality of individual genes on a genome-wide scale, the present study generated a large transposon mutant pool (input) that was subjected to *in vivo* selection (output). High-throughput Illumina sequencing technology was used to determine the exact position of transposon insertion site, and the number of reads at each insertion site in the input and output pools were then analyzed to identify the gene set important for MAP survival inside a mammalian host. Our data indicate that MAP-specific genes were under-represented in genes required for survival *in vitro* but over-represented among those predicted to contribute to survival *in vivo*, with both results highly statistically significant. Furthermore, our data identified MAP genes that are conserved across other mycobacterial species whose disruption resulted in an inability to survive *in vivo*, potentially offering candidate genes for the generation of live, attenuated vaccines.

Of the 4350 genes in the MAP genome, 2443 (56%) genes were disrupted by the transposon, indicating that we did not achieve 100% saturation in our input pool. While some of genes are presumably essential *in vitro*, and cannot be disrupted, our result fell short of expectations and suggested that we had incomplete disruption coverage of the genome. Although we harvested ~ 90000 clones for the input pool, we only achieved ~ 12000 unique transposon insertion mutants. This phenomenon was likely due to a bottlenecking effect during sample preparation or sequencing stage. This sparse disruption frequency prevented us from calling essential domains within a gene with statistical confidence. To address this issue in future studies, we will generate more independent libraries to maximally saturate the number of transposon insertions, which would allow us to study not only at the gene level but also intergenic regions and domains required for optimal growth under different conditions. Nonetheless, the assessment of these 2443 genes, including 82 MAP-specific genes, provides the first comprehensive portrait of genes required for survival of MAP, *in vitro* and *in vivo*. This method can now be readily applied to defined culture conditions that are deemed representative of the life cycle of MAP or to *in vivo* infections of the natural host, to test whether there are host-specific essential genes in the MAP genome.

MAP-specific genes are distributed on 6 genomic islands known as large sequence polymorphisms (LSP^P^s) [13]. These gene clusters are absent in *M. avium* subsp. *hominissuis* (MAH), the putative ancestor of MAP and a generally non-pathogenic strain [13], thus we were particularly interested in assessing whether the presence of these genes have increased MAP’s fitness as a professional pathogen. Indeed, we observed nearly twice as many LSP^P^ mutants to be depleted than expected after animal infection and our results indicated that these findings were not clustered to 1 island, but rather pertained to each of these 6 genome islands. Of note, different groups, using different comparison strains and technical platforms, have estimated the precise number of MAP-specific genes differently. Using the set of MAP-specific genes described by Castellanos *et al.*[33] which comprises 200 MAP-specific genes including the 96 LSP^P^ genes identified by Alexander *et al*. [13], we observed 160 disrupted genes, and 42 were depleted after the *in vivo* challenge. The enrichment was higher than expected (*p* value = 0.0012) and the odds ratio of MAP-specific gene disruption and *in vivo* attenuation was 1.82 in this case, showing a similar trend as our previous analysis.

Within LSP^P^4, MAP0856c shares no homology with any known protein; the closest orthologue of MAP0862 is found in *Acidothermus cellulolyticus*, a cellolytic thermophilic actinobacterium [34]. Of particular interest, the disruption of MAP0865 led to complete absence of mutants carrying this mutation in all outputs. MAP0865 is conserved in the cell division protein FtsK in *Streptomyces violaceusniger*. In *M. tuberculosis*, *ftsK* (*Rv2748c*) is essential for *in vitro* growth [19, 21] and has been predicted to be involved in cell division [32].

Genes found to be depleted in LSP^P^11 include: MAP2148, with a phage integrase orthologue in *Geodermatophilus obscurus*, a bacterium often found in stressful environments [35]; *MAP2150* and *MAP2157*, each likely encodes a transposase; MAP2154c and MAP2158 have no known function or orthologue in another organism.

Within LSP^P^12, *MAP2185c* was found to be important for *in vivo* growth; it shares homology with an amidohydrolase found in *Frankia*, a genus of bacteria that are nitrogen-fixing and often plant symbionts [36]. Another gene, *MAP2194* is part of the mammalian cell entry (mce) operon. In *M. tuberculosis*, the *mce* genes are known to facilitate mycobacterial cell entry and thus virulence factors [19, 37, 38]. The *mce* gene clusters are predicted to function as ATP-binding cassette (ABC) transporters for cholesterol [39–41], a substrate implicated in MAP pathogenesis [42].

LSP^P^14 constitutes the largest MAP-specific genomic island, and contains several blocks predicted to mediate functions such as metal acquisition and synthesis of metabolic and transport proteins [13]. In this study, *MAP3731c*, *MAP3733c,* and *MAP3735c* were found to be depleted in the output pool. They are part of an inorganic metal uptake functional unit that spans *MAP3731c* to *MAP3736c*. Strikingly the attenuated vaccine strain 316 F has been reported to have a deletion spanning *MAP3714-MAP3735c*[43]; this region has therefore been independently linked to *in vivo* survival by both gene deletion and Tn-induced gene disruption. In addition, *MAP3734c-3736c* have been found to be upregulated during bovine epithelial cells and macrophages [44] while a transcriptomic study found that *MAP3731-3736c* were downregulated in infected bovine tissues [45]. *MAP3740* to *MAP3746* has been predicted to be involved in siderophore biosynthesis; disruption in *MAP3741*, *MAP3742*, *MAP3745* all resulted in reduction in the output pool. As the first gene involved in mycobacterial siderophore (mycobactin) biosynthesis is truncated in MAP K-10 [14], it is of great interest to elucidate the function of this genetic element. Another set of depleted genes consisted of *MAP3750* and *MAP3751*, encoding membrane protein MmpS1 and MmpL4. Other depleted genes include *MAP3757c*, a probable leucyl-tRNA synthetase, *MAP3759* a transposase, MAP3760c a predicted methylase and two adjacent genes, *MAP3763c*, and *MAP3764c,* predicted to code for proteins involved in polyketide synthesis (PapA3 and Pks2 respectively) [46].

LSP^P^15 contains a putative metal uptake operon with a ferric uptake regulator (Fur)-like transcriptional regulator. In our study we identified disruption in *MAP3776c*, the first gene in this genomic region led to depletion in the output. *MAP3776c* encodes the solute-binding portion of an ABC transporter and is found to be downregulated in infected tissue [45]. The functional characterization of this operon is currently underway in our laboratory. Finally, LSP^P^16 contained two depleted genes, *MAP3816* which encodes a phage integrase, and *MAP3817c* which encodes a protein possibly involved in thiamine biosynthesis [47].

Compared to two previous studies that screened MAP transposon libraries for attenuated mutants, we also observed depletion (below 0.2 × median) of *MAP1694, MAP2231*, *MAP2232*, *MAP3963, MAP2205c*, *MAP3212*, and *MAP3607* in the output pool. Both studies used ATCC strain 19698 and different infection models (Balb/c mice and bovine kidney epithelial cells) [30, 31], and the consistency between these studies and our data suggests that these genes are very likely to be important for the survival of MAP in a mammalian host. Table 5 lists genes in which disruption resulted in complete absence in the output pool. A closer examination of these genes revealed that some of them are essential for *in vitro* growth in *M. tuberculosis*. A possible explanation is that our input pool was only passaged in rich growth medium (7H9) once, thus mutants with disruption in these essential genes were not completely eliminated but potentially growing poorly prior to infection.

To our knowledge, the present study is the first report that describes the assessment of conditionally important genes in MAP at a genome-wide scale. As MAP is a very slow-growing and fastidious microorganism, this transposon-mediated screen offers a powerful and unbiased tool for identifying the genetic basis for survival of MAP within a mammalian host. Further functional characterization of these promising candidates will undoubtedly shed light on the metabolism, genetic regulation, and virulence of MAP.

## Conclusions

The present study demonstrates that MAP-specific genes are over-represented in genes required for MAP to survive *in vivo*, but under-represented for its growth *in vitro*. Our finding provides support for the notion that horizontally transferred genetic elements specific to MAP contributed to its emergence as a professional pathogen. In addition, genes identified as essential for growth of MAP *in vitro* and *in vivo* present as potential targets for therapeutic development.

## Methods

### Bacteria and growth conditions

*Mycobacterium avium* subsp. *paratuberculosis* K-10 was used as the parental strain for transposon mutant library construction. Bacteria were grown with rotation at 37°C in Middlebrook 7H9 medium (Difco Laboratories, Detroit, MI) containing 0.2% glycerol, 0.1% Tween 80 (Sigma-Aldrich, St. Louis, MO), 10% albumin-dextrose-catalase (Becton Dickinson and Co., Sparks, MD), and 2 μg/ml of mycobactin J (Allied Monitor, IN). Transduction mutants were selected on Middlebrook 7H10 solid medium supplemented with 10% oleic acid-albumin-dextrose-catalase (Becton Dickinson and Co., Sparks, MD) and 50 μg/ml of kanamycin.

### Transposon insertion mutant library construction

Transposon library was generated as described [48]. Briefly, the MycoMarT7 phagemid was titered and amplified using *M. smegmatis* at 30°C. The phagemid contains the kanamycin-marked MycoMarT7 transposon that can be integrated into a TA dinucleotide site in the host DNA and has been extensively used to create high-density mutagenesis in mycobacteria [49]. *Mycobacterium avium* subsp. *paratuberculosis* at an OD_600_ of ~0.6 were transduced with ~3 × 10^9^ phages in MP buffer (50 mM Tris-HCl [pH 7.6], 150 mM NaCl, 2 mM CaCl_2_) for 4 hours at 37°C, transferred to 7H9 medium for 24 hours with rotation at 37°C, and subsequently plated on selective 7H10 medium. Kanamycin-resistant colonies (~8.8 × 10^4^) were evenly resuspended in 7H9 containing 25% glycerol and kanamycin, aliquoted and stored at -80°C until further use.

### Animals

C57BL/6 mice were purchased from Jackson Laboratories and maintained in a pathogen-free environment at the McGill University Health Centre. All animal experiments were in compliance with the regulations of the Canadian Council of Animal care and approved by the McGill University Animal Committee. Five mice were intraperitoneally injected with 0.74 × 10^8^ colony-forming units (CFUs) of transposon mutants. The inoculum was plated on 7H10 agar media for colony quantification as well as to study the input pool. One month after infection, the mice were sacrificed, and their spleens were aseptically removed, homogenized, and plated onto 7H10 kanaymcin plates to harvest surviving mutants (5 output pools).

### Genomic library preparation

High quality genomic DNA was extracted from input and output plates as described [50]. Subsequent DNA partial digestion, ligation to asymmetric adapters, transposon junction amplification, addition of Illumina sequencing sites by nested PCR were performed according to [21]. Amplified fragments between 250 – 400 base pairs were gel-purified and sequenced with generic Illumina primer (5′ACACTCTTTCCCTACACGACGCTCTTCCGATCT) using an Illumina HiSeq2000 system at the McGill University and Génome Québec Innovation Centre, and 100 base pair reads were generated.

### Sequence mapping and analysis

Transposon sequence up to the TA insertion site and regions of lower quality bases were trimmed off in all sequenced reads using a custom Python script. The sequences were aligned to the *M. avium* subsp. *paratuberculosis* K-10 reference genome [14] using Bowtie2 alignment software [51]. Reads aligned to multiple sites are assigned randomly to a mapped site. Aligned Sequence Alignment/Map (SAM) files were converted into binary BAM files using SAMtools [52]. Reads were then parsed and mapped to genomic coordinates of the TA sites using MATLAB® with custom scripts. For each TA insertion site, the number of reads detected and strand orientation were determined. Each insertion site coordinate was mapped to a protein coding gene or an intergenic region annotated in RefSeq file NC_002944.2.ptt (ftp://ftp.ncbi.nlm.nih.gov/genomes/Bacteria/Mycobacterium_avium_paratuberculosis_K_10_uid57699/NC_002944.gff). Insertion sites with ≤ 10 reads in the input pool were not considered in further analyses as we wished to test for relative depletion in the output compared to the input and needed a robust denominator as the basis for this comparison. The relative representation of each mutant after *in vivo* challenge was determined by calculating the ratio present in the output pool compared to the ratio present in the input pool (reads at each insertion/total reads in output divided by reads at each insertion/total reads in input). Read position was visualized by either Integrative Genomics Viewer (http://www.broadinstitute.org/igv/) [53, 54] or DNAPlotter (http://www.sanger.ac.uk/resources/software/dnaplotter/) [55] and multiple sites within a gene were then assessed together to generate estimates of essentiality as a function of genes. The output:input ratio of all disrupted insertion sites with more than 10 reads are listed in the Additional file 1. In addition, in all tables and supplemental data we also provided gene annotation generated by Li *et al.*[14] as well as the revised version by Wynne *et al.*[15] to improve accuracy as well as consistency for other researchers. Genes depleted in output pools are listed in Table 5 along with ortholog essentiality in *M. tuberculosis* and their putative functions [32, 56].

## Electronic supplementary material

Additional file 1: **Tn insertion data from input and output pools.** First tab: “All data” – region description, genomic position, total reads at each site with ≥ 11 reads aligned, proportion of each site relative to sequenced library, Output:Input ratio, and median value of ratio 1-4. Second tab: “0.2 × median” – insertion sites, genes (derived from insertion sites), and LSP^P^ genes depleted at 0.2 × median. Third tab: “0.1 × median” – insertion sites, genes (derived from insertion sites), and LSP^P^ genes depleted at 0.1 × median. Fourth tab: “0.05 × median” – insertion sites, genes (derived from insertion sites), and LSP^P^ genes depleted at 0.05 × median. For tabs 2-4, data were analyzed using LSP^P^ genes determined by Alexander *et al*[13]. For the fifth tab: “0.1 × median Castellanos et al” – insertion sites, genes (derived from insertion sites) and MAP-specific genes depleted at 0.1 × median, data were analyzed using MAP-specific genes identified by Castellanos *et al*[33]. (XLSX 2 MB)
